# Supplementary figures and images for: Chicoric Acid Ameliorated Beta-Amyloid Pathology and Enhanced Expression of Synaptic-Function-Related Markers via L1CAM in Alzheimer’s Disease Models
Source: Int J Mol Sci. 2024 Mar 17;25(6):3408. doi: 10.3390/ijms25063408 (PMC10970381; doi:10.3390/ijms25063408)

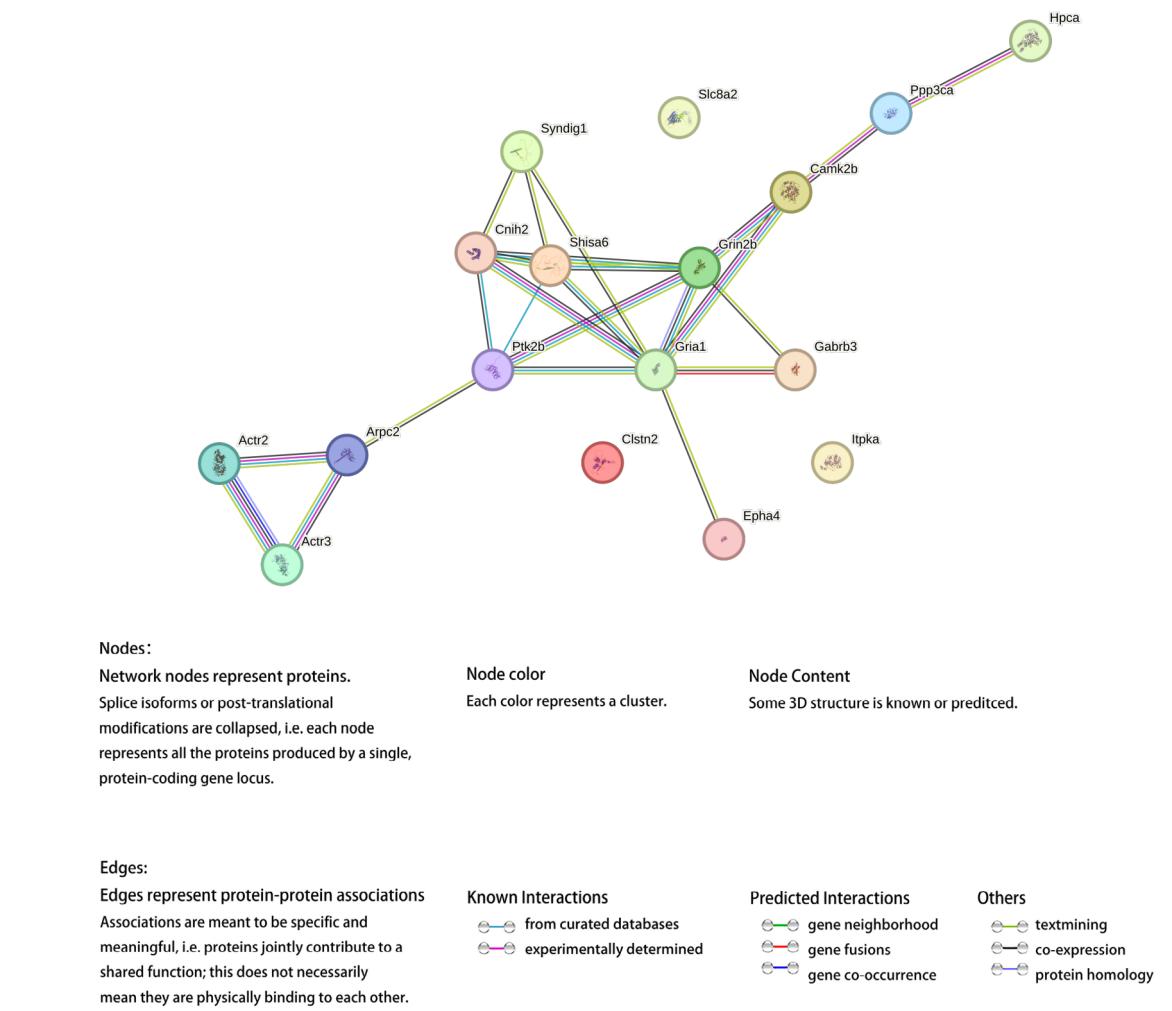

Supplement: Supplementary file 1 [file ijms-25-03408-s001.zip › FIG S1.jpg]

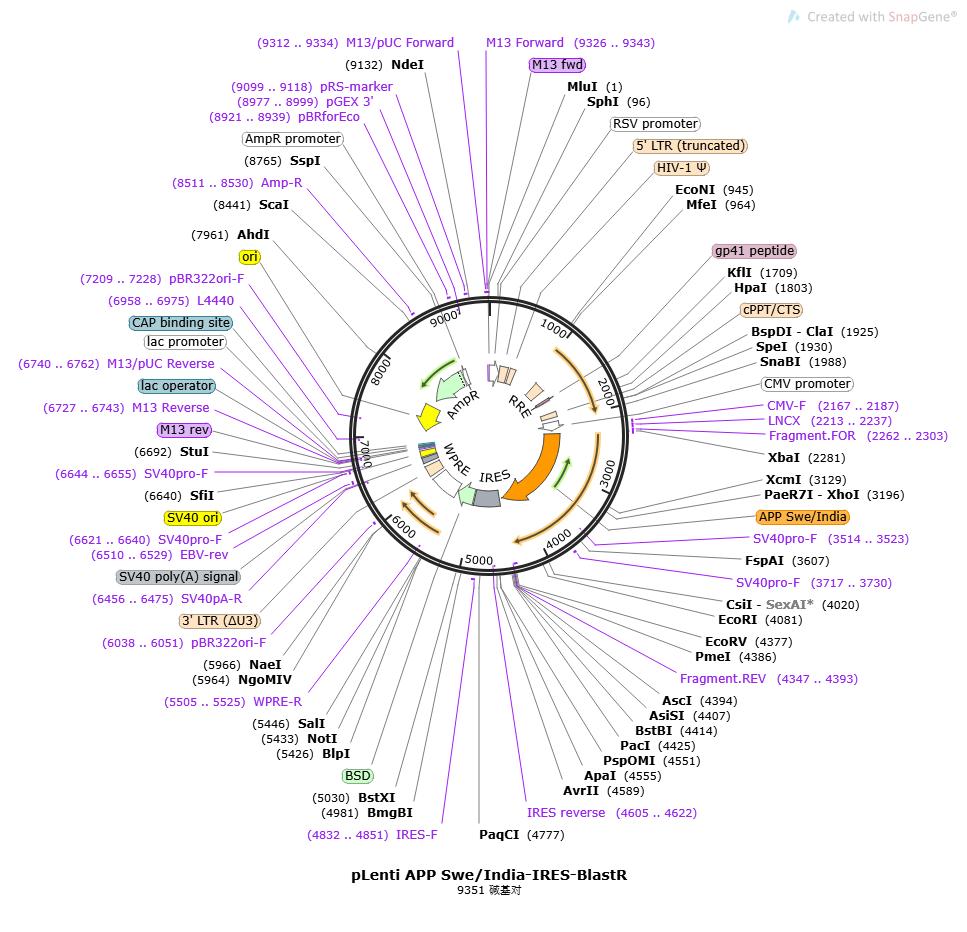

Supplement: Supplementary file 1 [file ijms-25-03408-s001.zip › FIG S2.jpg]

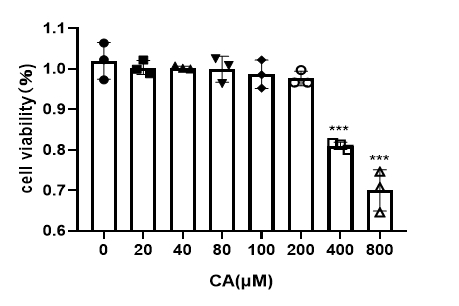

Supplement: Supplementary file 1 [file ijms-25-03408-s001.zip › FIG S3.jpg]
